# Vascular endothelial growth factor receptor-1 mediates migration of human colorectal carcinoma cells by activation of Src family kinases

**DOI:** 10.1038/sj.bjc.6603143

**Published:** 2006-05-09

**Authors:** D P Lesslie, J M Summy, N U Parikh, F Fan, J G Trevino, T K Sawyer, C A Metcalf, W C Shakespeare, D J Hicklin, L M Ellis, G E Gallick

**Affiliations:** 1 Department of Surgical Oncology, The University of Texas MD Anderson Cancer Center, 1515 Holcombe Boulevard, Houston, TX, USA; 2 Department of Cancer Biology, 1515 Holcombe Boulevard, Houston, TX, USA; 3 Ariad Pharmaceuticals, Cambridge, MA, USA; 4 ImClone Systems, New York, NY, USA

**Keywords:** VEGFR-1, VEGF, Src kinase, colorectal cancer

## Abstract

Vascular endothelial growth factor (VEGF) is the predominant pro-angiogenic cytokine in human malignancy, and its expression correlates with disease recurrence and poor outcomes in patients with colorectal cancer. Recently, expression of vascular endothelial growth factor receptors (VEGFRs) has been observed on tumours of epithelial origin, including those arising in the colon, but the molecular mechanisms governing potential VEGF-driven biologic functioning in these tumours are not well characterised. In this report, we investigated the role of Src family kinases (SFKs) in VEGF-mediated signalling in human colorectal carcinoma (CRC) cell lines. Vascular endothelial growth factor specifically activated SFKs in HT29 and KM12L4 CRC cell lines. Further, VEGF stimulation resulted in enhanced cellular migration, which was effectively blocked by pharmacologic inhibition of VEGFR-1 or Src kinase. Correspondingly, migration studies using siRNA clones with reduced Src expression confirmed the requirement for Src in VEGF-induced migration in these cells. Furthermore, VEGF treatment enhanced VEGFR-1/SFK complex formation and increased tyrosine phosphorylation of focal adhesion kinase, p130 cas and paxillin. Finally, we demonstrate that VEGF-induced migration is not due, at least in part, to VEGF acting as a mitogen. These results suggest that VEGFR-1 promotes migration of tumour cells through a Src-dependent pathway linked to activation of focal adhesion components that regulate this process.

Three distinct receptor tyrosine kinases bind vascular endothelial growth factor (VEGF): vascular endothelial growth factor receptor (VEGFR)-1 (Flt-1), VEGFR-2 (Flk-1/KDR) and VEGFR-3 (Flt-4). Vascular endothelial growth factor receptor 1 and VEGFR-2 are expressed primarily on vascular endothelial cells, while VEGFR-3 is expressed primarily on lymphatic endothelial cells and regulates lymphangiogenesis (Irrthum *et al*, 2000). Vascular endothelial growth factor receptor 2 is believed to be the major mediator of angiogenesis in human malignancy, as it regulates activation of downstream effector molecules such as phosphoinositide 3-kinase and AKT, and potentiates endothelial differentiation, DNA synthesis and proliferation (Waltenberger *et al*, 1994; Meyer and Rahimi, 2003). Vascular endothelial growth factor receptor 1 appears to function as a VEGF ‘sink’ during developmental vasculogenesis, but may contribute to angiogenesis in pathologic states such as ischaemia or malignancy (Hiratsuka *et al*, 1998, 2001; Carmeliet *et al*, 2001; Hayashibara *et al*, 2001).

In addition to their expression on endothelial cells, VEGFRs are also expressed on cells of haematopoietic origin and have recently been demonstrated on a variety of tumour types, including prostate, ovarian, melanoma, non-small-cell lung, pancreatic and colon (Decaussin *et al*, 1999; Ferrer *et al*, 1999; Masood *et al*, 2001). While the functions of VEGFRs on tumour cells are not completely understood, the concomitant production of VEGF and VEGFR expression by tumour cells suggests the possibility that these receptors mediate biologic functions in tumour cells. Indeed, in cancers of the breast and skin (melanoma) and in some leukaemias, VEGF/VEGFR autocrine signalling loops have been identified (Bellamy *et al*, 1999; Dias *et al*, 2000, 2001; Lacal *et al*, 2000), but the elucidation of the contribution of individual VEGF/VEGFR family members to biologic functions mediated by individual receptors is only beginning to emerge. In a recent work, Wang *et al* (2004) demonstrated differential regulation of lymphoma xenografts utilising species-specific receptor antibodies to VEGFR-1 and VEGFR-2. In that study, targeting tumour-associated VEGFR-1 (human xenografted cells) increased apoptosis and diminished tumour growth, while targeting host (i.e. murine) VEGFR-2 diminished microvascular density (Wang *et al*, 2004). Also, inhibition of VEGFRs with a synthetic binding antagonist inhibited growth in xenograft models of colon and other tumours (Ueda *et al*, 2005). Individual receptor ligands may elicit different biologic responses as well. In *PlGF*−/− mice, VEGF-B (another VEGFR-1 ligand) failed to rescue vascular development, suggesting that differential responses occur depending on which ligand binds the receptor *in vivo* (Carmeliet *et al*, 2001).

Signalling pathways mediated by VEGFRs in both endothelial and tumour cells are now being delineated. In endothelial cells, the Src family kinases (SFKs), Src and Yes are required for VEGF-induced vascular permeability and survival (Eliceiri *et al*, 1999). Likewise, VEGF-induced Src activation and signalling has been reported in Kaposi's sarcoma cells (Munshi *et al*, 2000). However, the possibility that VEGFRs require SFKs to mediate their biologic effects in other tumours has not been established.

When bound to receptor protein tyrosine kinases, SFKs become activated. Activation of SFKs has been implicated in progression and metastasis of a variety of solid tumours (reviewed by Summy and Gallick, 2003). Experiments in colorectal cancer have demonstrated increasing Src kinase activity with progression from adenoma to dysplasia to carcinoma and, finally, to metastatic disease (Talamonti *et al*, 1993), suggesting a role for Src in regulating colon tumour progression. Additionally, Src kinase activity and VEGF have both been associated with poor prognosis in patients with advanced colorectal cancer (Takahashi *et al*, 1997; Werther *et al*, 2000; Allgayer *et al*, 2002). Thus, the potential of VEGFRs to signal through SFKs in colorectal cancer may be of clinical significance.

The purpose of this study was to determine whether SFKs must be activated in order for VEGFRs to mediate biologic effects. We tested the effects of VEGFR signalling through SFKs on cellular migration and proliferation in human colorectal cancer cells. We found that migration, but not proliferation, was significantly increased, suggesting the potential role of VEGFR-1 expression in tumour progression in these cells.

## MATERIALS AND METHODS

### Cell culture

HT29 cells derived from a human colon adenocarcinoma were maintained as a subconfluent monolayer in Dulbecco's modified Eagle's medium (DMEM/F12) with Earle's salts and 2 mM glutamine (Life Technologies, Inc., Grand Island, NY, USA) supplemented with 10% foetal bovine serum (Hyclone Laboratories, Logan, UT, USA) without antibiotics and incubated in 5% CO_2_/95% air at 37°C. Highly metastatic KM12L4 human colorectal carcinoma (CRC) cells were kindly provided by IJ Fidler, PhD, DVM (The University of Texas MD Anderson Cancer Center, Houston, TX, USA). Cells were cultured and maintained in minimal essential medium (MEM) supplemented with 10% foetal bovine serum (Hyclone Laboratories), 2 U ml^−1^ of a penicillin–streptomycin mixture (Flow Laboratories, Rockville, MD, USA), vitamins (Life Technologies, Inc., Grand Island, NY, USA), 1 mM sodium pyruvate, 2 mM L-glutamine and nonessential amino acids, and incubated in 5% CO_2_/95% air at 37°C.

### Creation of siRNA expression plasmids silencing Src gene expression

SiRNA expression plasmids were created using the Ambion pSilencer 1.0-U6 (Austin, TX, USA) according to the manufacturer's directions. c-Src specific target sequences were designed using the Ambion siRNA web design tool. The two target sequences utilised were (52–71 bp) 5′-AACAAGAG CAAGCCCAAGGAT-3′ and (226–244 bp) 5′-AAGCTGTTCGGAGGCTTCAAC-3′. Oligonucleotides corresponding to these sequences with flanking *Apa*1 (5′) and *Eco*R1 (3′) ends were purchased from Invitrogen/Life Technology (Carlsbad, CA, USA) and ligated into the expression plasmid at compatible sites. Constructs were confirmed by DNA sequencing. HT29 cells were then transfected with 500 ng of each siRNA plasmid and 100 ng of pcDNA G418 resistance promoter-less plasmid for selection of transfectants. Cells were then grown in selective media containing G418 as described previously (Ahmad *et al*, 2001). Negative controls were transfected with empty vector target sequences and pcDNA plasmids at identical concentrations. Total c-Src expression levels in siRNA clones were determined by Western blot analysis.

### Inhibitors/recombinant growth factors

The novel, selective, potent Src kinase inhibitor, AP23464 (O'Hare *et al*, 2004; Brunton *et al*, 2005; Corbin *et al*, 2005; Summy *et al*, 2005), or the commercially available Src kinase inhibitor, 4-amino-5-(4-chlorophenyl)-7-(*t*-butyl)pyrazolo(3,4-d)pyrimidine (PP2) (Calbiochem, San Diego, CA, USA) suspended in 1% dimethyl sulphoxide (DMSO) at desired concentrations and the VEGFR-1 monoclonal blocking antibody IMC-18F1 were used in this study. The recombinant human growth factors VEGF-A and VEGF-B (R&D Systems, Minneapolis, MN, USA) were used at 10 and 50 ng ml^−1^, respectively, unless noted otherwise.

### Cell lysis

Cells in log growth phase at 50% confluency were serum starved overnight and exposed to the VEGFR-1 blocking antibody IMC-18F1 (20 *μ*g ml^−1^), or AP23464 (1 *μ*M) was added to culture media for 1 h at 37°C. Recombinant VEGF-A or -B or PBS control was then added at the desired concentration, and, at the desired time, cells were rinsed twice with ice-cold PBS and then lysed with RIPA-B lysis buffer (20 mM sodium phosphate, 150 mM NaCl, 5 mM EDTA, 1% Triton X-100, 0.5% sodium deoxycholate) supplemented with one tablet complete mini-EDTA protease inhibitor cocktail (Roche Diagnostic, Manheim, Germany) and 1 mM sodium orthovanadate (pH 7.4). Cells were harvested with the aid of a rubber policeman, clarified by centrifugation at 13 000 **g** for 15 min at 4°C, and prepared for Western blot analysis or immunoprecipitation, as described previously (Windham *et al*, 2002).

### Immunodepletion assay

Cleared cell lysates (250 *μ*g protein) were incubated overnight at 4°C with 10 *μ*l anti-VEGFR-1 antibody (Oncogene Research Products, Cambridge, MA, USA) or 10 *μ*l antimouse IgG (Organon Teknika, Durham, NC, USA). Immune complexes were formed by the addition of 50 *μ*l protein A:G agarose beads for 2 h at 4°C. The remaining soluble lysates were centrifuged at 13 000 **g** for 2 min and the supernatants were collected for immunoblotting.

### Immunoprecipitation and immune complex kinase assay

Cleared cell lysates (500 *μ*g protein) were incubated overnight at 4°C with 10 *μ*l of Src monoclonal antibody 327 (Oncogene Research Products, Cambridge, MA, USA), 10 *μ*l Yes antibody 1B7 (WAKO Biologicals, Richmond, VA, USA) or 6 *μ*l focal adhesion kinase (FAK) antibody (clone 2A7, Upstate Biotechnology, Lake Placid, NY, USA). Immune complex kinase assays were performed as described previously (Windham *et al*, 2002). Briefly, immune complexes were formed by the addition of 10 *μ*l of rabbit antimouse IgG (Organon Teknika, Durham, NC, USA) for 2 h, followed by 50 *μ*l of 10% (v v^−1^) formalin-fixed Pansorbin (*Staphylococcus aureus*, Cowan strain; Calbiochem, La Jolla, CA, USA) for 60 min. Pellets were then washed three times in a buffer consisting of 0.1% Triton X-100, 150 mM NaCl and 10 mM sodium phosphate. Reactions were initiated at 22°C by the addition of 10 *μ*Ci of [*γ*^32^P]ATP, 10 mM Mg^2+^ and 100 *μ*M sodium orthovanadate in 20 mM HEPES buffer to each sample. To analyse phosphorylation of an exogenous substrate, 10 *μ*g of rabbit muscle enolase (Sigma-Aldrich, St Louis, MO, USA) was added to the reaction buffer. After 10 min, reactions were terminated by the addition of SDS sample buffer. Proteins were separated by SDS–PAGE on 8% polyacrylamide gels, and radioactive proteins were detected by autoradiography.

### Immunoblotting

Proteins (50 *μ*g) from clarified cell lysates were separated via 8% SDS–PAGE and electroblotted onto polyvinylidene difluoride membranes (Amersham Corp., Chicago, IL, USA). The membranes were blocked with Tris-buffered saline/Tween (0.15)+5% dried milk for 30 min at room temperature and probed with the desired primary antibody diluted 1 : 1000 in blocking buffer overnight at 4°C. Membranes were probed with antibodies to Src mAb#327 (Oncogene Research Products), Yes Ab 1B7 (WAKO Biologicals), phospho FAK^Y397^ (Biosource International, Camarillo, CA, USA), phospho FAK^Y861^ (Biosource International), FAK (BD Transduction, San Jose, CA, USA), Akt (5G3, Cell Signaling Technology, Beverly, MA, USA), phospho-Akt^S473^ (Cell Signaling Technology), p42/44 Erk MAPK (Cell Signaling Technology), phospho-p42/44 Erk^T202/Y204^ (Cell Signaling Technology), VEGFR-1 (Oncogene Research Products), paxillin (Cell Signaling Technology), phospho-paxillin^Y118^ (Cell Signaling Technology), p130^cas^ (BD Transduction), phospho-p130^cas/Y165^ (Cell Signaling Technology), phosphotyrosine (4G10, Upstate, Lake Placid, New York, NY, USA) and vinculin (Sigma-Aldrich). Primary antibody incubation was followed by incubation with a horseradish peroxidase-conjugated secondary antibody (Bio-Rad goat anti-mouse, sheep anti-rabbit or rabbit anti-goat; Bio-Rad laboratories, Hercules, CA, USA) diluted 1 : 3000 in blocking buffer for 1 h at room temperature. Proteins were visualised with electrochemiluminescence detection reagents (Perkin-Elmer, Boston, MA, USA) and detected with autoradiography.

### Proliferation assay

3-(4,5-Dimethylthiazol-2-yl)-2,5-diphenyltetrazolium bromide (M2128) (Sigma Chemical Corp., St Louis, MO, USA) was prepared by dissolving 5 mg M2128 in 1 ml PBS. The solution was protected from light and stored at 4°C. To determine proliferation, HT29 cells (1.5 × 10^3^ cells) were seeded into 96-well plates in quintuplicate and allowed to adhere overnight in 10% complete DMEM/F12. The medium was then removed and replaced with 0.2 ml of media containing VEGF-A supplemented with solvent control, IMC-18F1 or AP23464 and allowed to incubate at 37°C. At the desired time point, 50 *μ*l of prepared MTT solution was added to each well and incubated at 37°C for 2 h. The media was removed carefully and the cells were solubilised in 0.2 ml DMSO. Plates were read using spectrophotometric analysis at a wavelength of 570 nm using the TECAN Genios plate reader and Magellan version 4.0 software. Results are representative of three independent experiments.

### Migration assay

The modified Boyden chamber migration assay was used as described previously (Minard *et al*, 2005). Cells (2.5 × 10^5^ cells) were suspended in the upper well of the migration chamber (control inserts, 8 *μ*m pore size; Becton-Dickinson, Bedford, MA, USA) in 0.5 ml of serum-free media. The lower chamber was filled with 0.75 ml of media with VEGF-A (10 ng ml^−1^) supplemented with DMSO control, IMC-18F1 (20 *μ*g ml^−1^) or AP23464 (1 *μ*M). After 72 h of incubation, the nonmigratory colon carcinoma cells on the upper filter surface were removed with a cotton swab, and cells that had migrated to the lower filter were fixed and stained with HEMA 3 (Biochemical Sciences, Swedesboro, NJ, USA) according to the manufacturer's instructions. The migratory cells were counted under a microscope at × 100 magnification. Cell images were obtained using a Sony PXC-990 3CCD colour video camera (Sony of America, New York, NY, USA). Cells were counted in five random fields per insert in triplicate.

### Statistical analyses

Statistical differences among groups were examined using the two-tailed Student's *t*-test. *P*<0.05 was considered statistically significant.

## RESULTS

### VEGF induces SFK activation in human CRC cells

**Figure 1:**
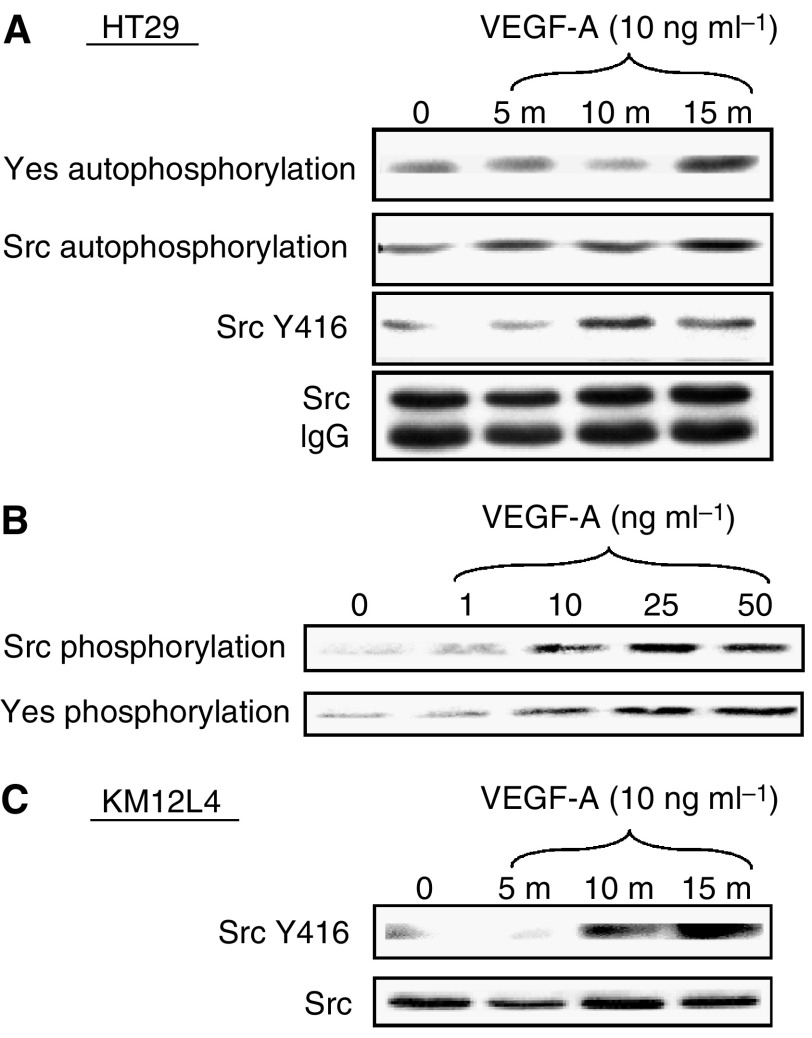
Effect of VEGF on SFK activity in human CRC cell lines. (**A**) Serum-starved HT29 cells at 50% confluency were untreated (0) or stimulated with VEGF-A for 5, 10 and 15 min. Cell lysates were immunoprecipitated with anti-Src or anti-Yes antibodies and subjected to immune complex kinase assays (top two panels) or Western blotting with the phospho-specific anti-Src Y416 antibody. Western blotting (following immunoprecipitation) with anti-Src antibody demonstrates equivalent total Src expression (bottom panel). (**B**) Serum-starved HT29 cells at 50% confluency were untreated (0) or stimulated with indicated concentrations of VEGF-A for 15 min. Cell lysates were immunoprecipitated with anti-Src or anti-Yes antibodies and subjected to immune complex kinase assay. (**C**) KM12L4 cell lysates were prepared and immunoblotted with SrcY416 antibodies and stripped and reprobed with total Src antibodies as described above.

A variety of growth factors are known to induce SFK activation in endothelial cells. We examined HT29 and KM12L4 human CRC cells to determine whether Src and Yes, the SFK members principally expressed in these cells (Cartwright *et al*, 1989; Park *et al*, 1993), are activated by VEGF-A. As measured by immune complex kinase assay, treatment of HT29 cells with VEGF-A (10 ng ml^−1^) increased both Src and Yes kinase activity in a time-dependent manner (Figure 1A). Maximal activation (∼2.5-fold increase) was observed within 15 min, which is consistent with Src activation by a number of ligands. Src expression did not change during the time period analysed, consistent with our postulate that VEGF-A increases specific activity of these SFKs. As shown in Figure 1B, VEGF-A also activates SFKs in a dose-dependent manner in HT29 cells, with maximal kinase activation observed at 25 ng ml^−1^. Likewise, treatment of KM12L4 human CRC cells with VEGF-A (10 ng ml^−1^) induced a similar increase in SFK activation (Figure 1C) as that observed in HT29 cells.

### VEGF-induced SFK activation occurs through VEGFR-1 in HT29 cells

**Figure 2:**
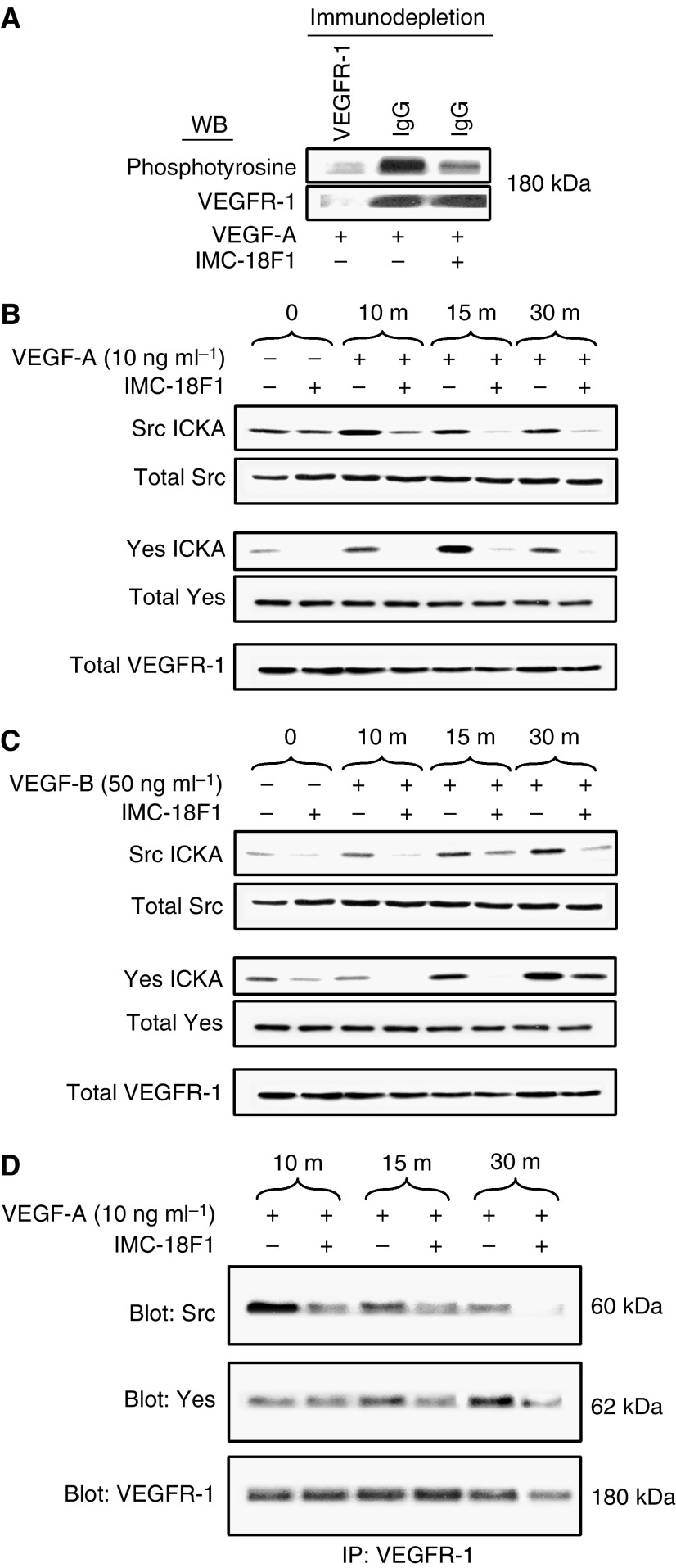
Requirement for VEGFR-1 for Src activation by VEGF-A and VEGF-B. (**A**) Lysates from VEGF-treated cells in the presence or absence of the VEGFR-1 blocking antibody, IMC-18F1, following immunodepletion with anti-VEGFR-1 antibody (first lane) or nonspecific IgG (second and third lanes) were prepared, and the resultant supernatants were subjected to Western blotting with anti-phosphotyrosine antibody or anti-VEGFR-1 antibody. (**B**, **C**) Serum-starved HT29 cells at 50% confluency were pretreated with IMC-18F1 or PBS control for 1 h and were untreated (0) or stimulated with VEGF-A or VEGF-B for 10, 15 and 30 min. Cell lysates were immunoprecipitated with anti-Src or anti-Yes antibodies and subjected to immune complex kinase assay or subjected to Western blotting with anti-Src, anti-Yes or anti-VEGFR-1 antibodies to demonstrate the lack of effect on expression of these proteins. (**D**) Lysates from VEGF-A-treated cells in the presence or absence or IMC-18F1 were immunoprecipitated with anti-VEGFR-1 antibody. The resultant immunoprecipitates were run on SDS–PAGE and subjected to Western blotting with antibodies to Src, Yes or VEGFR-1.

As reported previously, HT29 cells express VEGFR-1 but not VEGFR-2 or VEGFR-3 (Fan *et al*, 2005). To demonstrate VEGFR-1 activation and specificity of the monoclonal VEGFR-1 blocking antibody, IMC-18F1, tyrosine phosphorylation of VEGFR-1 was determined by Western blotting. As presented in Figure 2A, VEGF-A treatment resulted in marked increase in tyrosine phosphorylation of a 180 kDa cellular protein, which was effectively blocked by pretreatment with IMC-18F1, confirming the ability of IMC-18F1 to inhibit VEGFR-1 activation without altering VEGFR-1 expression levels. Next, to determine whether activation of SFKs was mediated by VEGFR-1, SFK activity with VEGF-A (ligand for both VEGFR-1 and -2) and the VEGFR-1 specific ligand VEGF-B was examined in the presence or absence of IMC-18F1. As shown in Figure 2B and C, VEGF-A and -B induced similar SFK activation at the doses indicated; however, pre-incubation of HT29 cells with IMC-18F1 (20 *μ*g ml^−1^) abrogated Src and Yes activation by both VEGF-A and -B, demonstrating that functional VEGFR-1 is required for VEGF-mediated SFK activation.

Increased activity of SFKs usually results from direct association with the cognate receptor. To determine whether VEGF induces SFK/VEGFR-1 complexes, co-immunoprecipitation experiments were performed with VEGF stimulation in the presence or absence of VEGFR-1 blocking antibody. As demonstrated in Figure 2D, VEGF greatly enhanced SFK/VEGFR-1 complex formation, and this association was effectively blocked by pretreatment with IMC-18F1. Immune complexes were not detectable when using an irrelevant IgG antibody (data not shown). Taken together, these results suggest that increased SFK activity may result from direct interaction with VEGFR-1, though other mechanisms might account for increased Src activity as well.

### VEGF induces migration of human CRC cells

**Figure 3:**
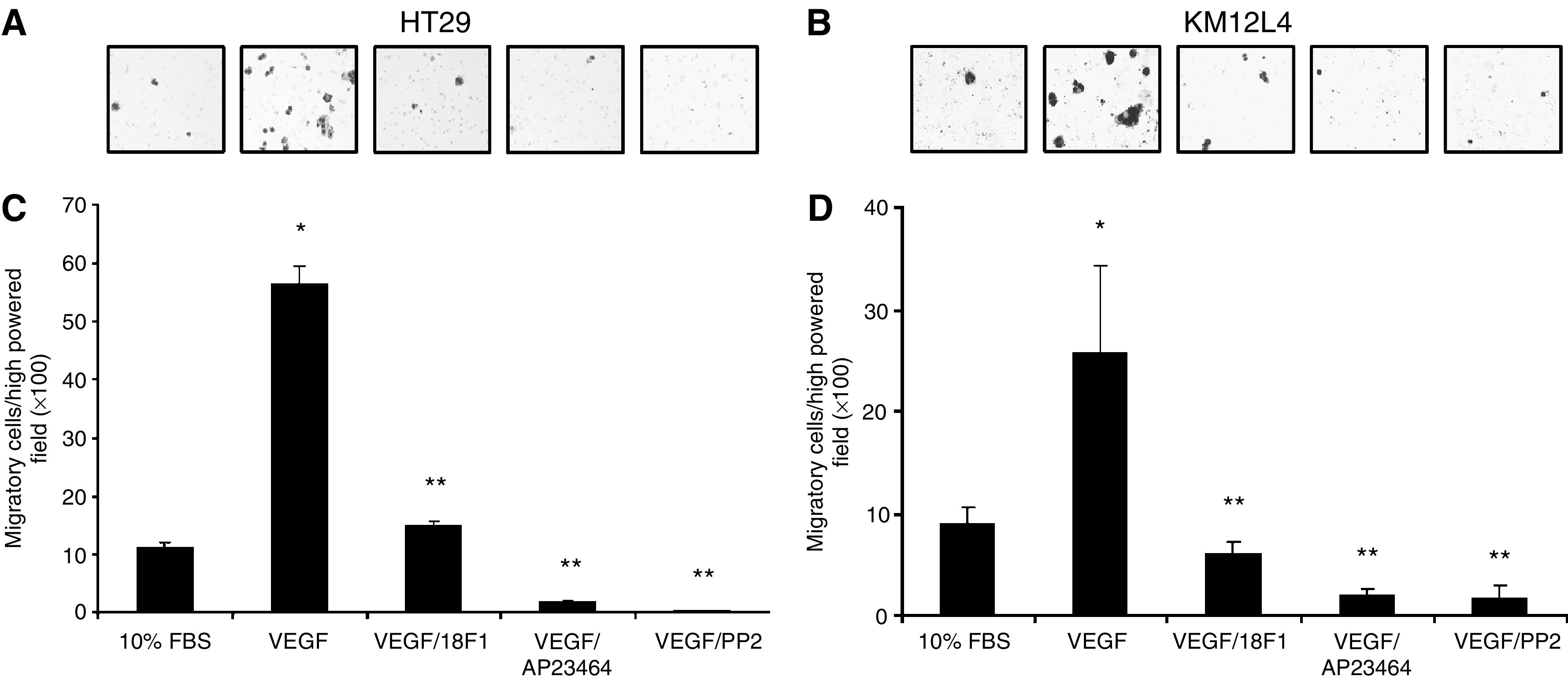
Effects of VEGF-mediated SFK activation on CRC migration. HT29 and KM12L4 CRC cells were pretreated with IMC-18F1 (20 *μ*g ml^−1^), the novel, potent Src kinase inhibitor, AP23464 (1 *μ*M), a commercially available Src inhibitor, PP2 (10 *μ*M) or PBS control for 1 h and allowed to migrate in a modified Boyden chamber containing VEGF-A (10 ng ml^−1^) or 10% FBS for 72 h. (**A**, **B**) Representative photos of migrated cells ( × 100 magnification). (**C**, **D**) Quantitation of migrated cells. ^*^P<0.001 *vs* control cells. ^**^P<0.001 *vs* cells treated with VEGF alone. Bars represent s.e.m.

Src family members have been implicated in numerous biologic activities, including proliferation and migration (reviewed by Brown and Cooper, 1996; Thomas and Brugge, 1997). To identify the potential biologic effects mediated by VEGFR-1 expression, we first assessed cellular migration in response to VEGF-A, utilising a modified Boyden chamber as described in Materials and Methods (Figure 3). As demonstrated in Figures 3B and D, VEGF-A treatment of HT29 and KM12L4 human CRC cells resulted in a five- and three-fold increases in cellular migration, respectively, over nontreated control cells (*P*<0.001). Pharmacologic blockade of VEGFR-1 with IMC-18F1 or Src kinase inhibition with AP23464 or PP2 completely inhibited VEGF-induced migration in both cell lines (*P*<0.001), again demonstrating the requirement of SFK activation through VEGFR-1 in this process.

### Effects of Src-targeted siRNA on VEGF-induced migration of CRC

**Figure 4:**
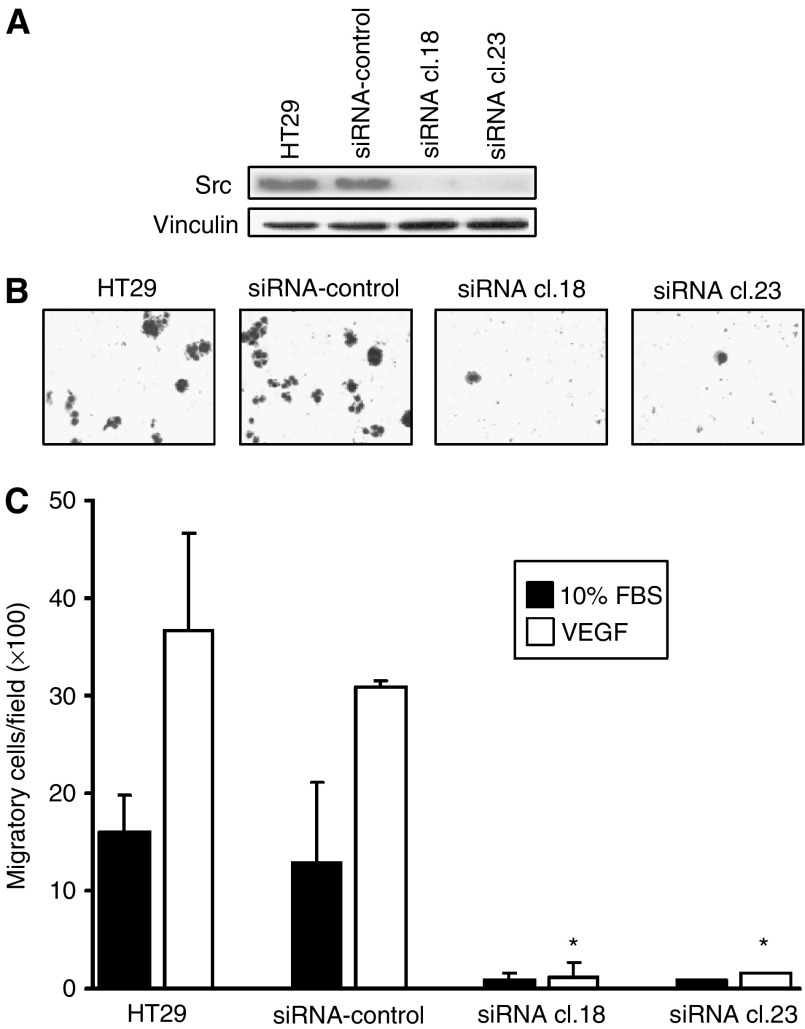
Effects of Src-targeted siRNA on VEGF-induced CRC migration. (**A**) HT29 parental cells and stable G418-resistant clones expressing either empty vector (siRNA control) or Src-targeted siRNA were subjected to Western blot analysis with antibodies to total Src. Membranes were stripped and reprobed with anti-vinculin antibody as a loading control. Parental HT29, siRNA control, siRNA cl. 18 and siRNA cl. 23 cells were placed in a modified Boyden chamber containing VEGF-A (10 ng ml^−1^) or 10% FBS for 72 h. (**B**) Representative photos of VEGF-A-treated cells ( × 100 magnification). (**C**) Quantitation of migrated cells. ^*^P<0.001 *vs* VEGF-treated siRNA control.

To independently confirm the requirement for Src in mediating VEGF-A-induced migration, the ability of this ligand to affect migration in HT29 clones reduced in Src by stable expression of an antisense expression vector was determined. As shown in Figure 4A, two independent clones (siRNA cl.18 and 23) were reduced by more than 80% in Src expression. These cells were considerably reduced in their migratory abilities (Figure 4B), consistent with Src being important in cellular migration, and addition of VEGF-A did not increase migratory capability of these cells (Figure 4C), providing further evidence that VEGF mediates migration through Src activation. Basal proliferation of these cells as determined by MTT assay did not differ significantly from nontransfected parental cells (data not shown).

### VEGF activates FAK, p130^cas^ and paxillin in HT29 cells

**Figure 5:**
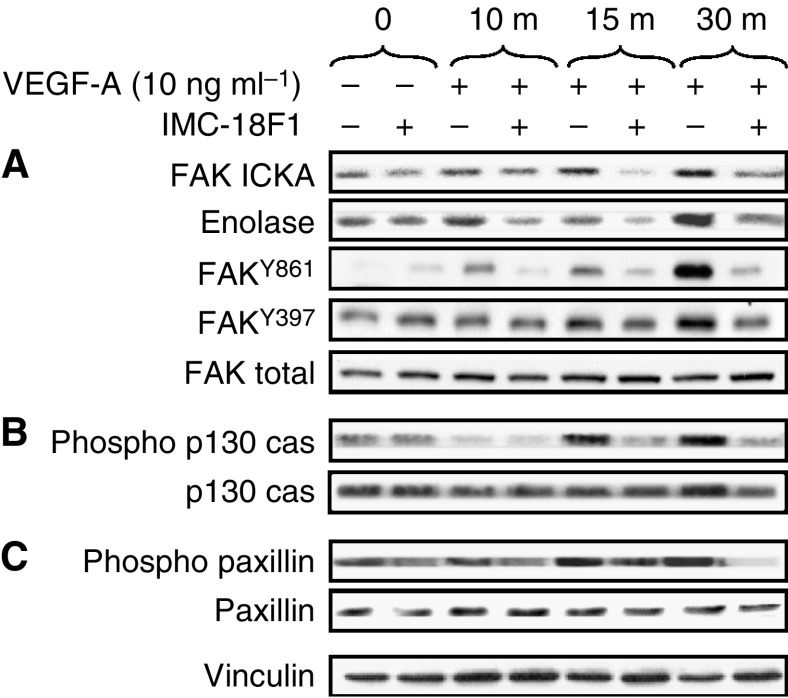
Effects of VEGF on phosphorylation of FAK, p130^cas^ and paxillin in CRC. Serum-starved HT29 cells at 50% confluency were pretreated with the VEGFR-1 blocking antibody (IMC-18F1) or PBS control for 1 h and were untreated (0) or stimulated with VEGF-A for 10, 15 and 30 min. Cell lysates were immunoprecipitated with anti-FAK antibody and subjected to immune complex kinase assay with enolase as an exogenous substrate or subjected to Western blotting with anti-phospho-FAK Y861, anti-phospho-FAK Y397 or anti-FAK antibodies as indicated (**A**), run on SDS–PAGE and subjected to Western blotting with anti-phospho-p130^cas^ or anti-p130^cas^ antibodies (**B**) or subjected to Western blotting with anti-phospho-paxillin or anti-paxillin antibodies (**C**). Western blot for vinculin is included to demonstrate equivalent protein loading.

In epithelial and fibroblast cells, migration is regulated, in part, by activation of FAK. Recent studies in endothelial cells have implicated FAK as required for VEGFR-1-induced tubulogenesis (Maru *et al*, 2001). Src/FAK activation then leads to phosphorylation of both paxillin and p130^cas^. To determine if FAK were activated upon treatment of HT29 cells with VEGF, both FAK immune complex kinase assays and Western blot analysis for specific FAK phosphorylation sites were performed as described in Materials and Methods. As presented in Figure 5A, VEGF treatment of HT29 cells increased both autophosphorylation of FAK and phosphorylation of the exogenous substrate enolase two-fold at 30 min. As enolase phosphorylation may also result from Src being immunoprecipitated in the immune complexes, we directly examined phosphorylation of Y861 and Y397 in response to VEGF stimulation of HT29 cells. Phosphorylation of Y861, and to a lesser extent Y397, was increased, and these increases were blocked by prior addition of IMC-18F1. These findings are consistent with previous experimental work in VEGFR-1 overexpressing fibroblasts (Maru *et al*, 2001) and suggest crosstalk between VEGFR-1 and FAK in HT29 cells. As shown in Figure 5B and C, VEGF treatment of HT29 cells also increased tyrosine phosphorylation of both paxillin and p130^cas^. Maximal phosphorylation occurred within 15–30 min, consistent with the kinetics of Src and Yes activation. Pretreatment of HT29 cells with IMC-18F1 effectively blocked FAK, paxillin and p130^cas^ phosphorylation, confirming the requirement of VEGFR-1 for VEGF-induced activation of these substrates. Together, these results suggest that a VEGFR-1/SFK complex interacts with components of focal adhesions, thus mediating cellular migration in HT29 cells.

### VEGF does not induce proliferation in HT29 cells

**Figure 6:**
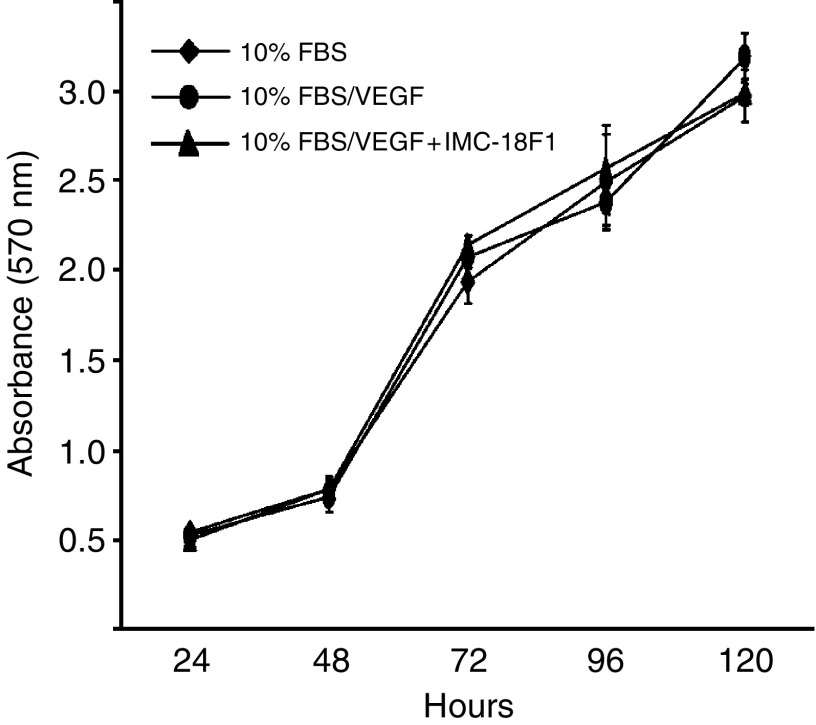
Effects of VEGF on CRC proliferation. HT29 cells (2 × 10^3^) in 96-well plates in serum-free media were untreated or treated with VEGF-A (10 ng ml^−1^) in the presence of AP23464 (1 *μ*M) or IMC-18F1 (20 *μ*g ml^−1^) and proliferation was determined by MTT assay as described in Materials and Methods. Bars represent standard error of the mean. Results are representative of three independent experiments.

Finally, to determine if VEGF-induced migration could be accounted for, at least in part, by VEGF acting as a mitogen, we examined the effect of VEGF-A stimulation on proliferation of HT29 cells. As shown in Figure 6, exogenous VEGF-A had no effect on HT29 cell proliferation, as determined by MTT assay. Likewise, pretreatment of HT29 cells with IMC-18F1 did not decrease proliferation. Consistent with these findings, VEGF-A treatment of HT29 cells induced minimal to no activation of Erk 1/2 or Akt, as assessed by Western blotting for phosphorylated (active) forms of these signalling enzymes (data not shown).

## DISCUSSION

Our findings of VEGF-induced SFK activation and enhanced cellular migration in human CRC cells demonstrate that functional VEGFR-1 mediates intracellular signalling and biologic behaviour in these cells. Src family kinase activation in response to VEGF has been observed in cells of endothelial origin expressing VEGFRs, and SFK members have been shown to associate with VEGFRs upon activation (Chou *et al*, 2002). In contrast to work in endothelial cells where Src preferentially associates with VEGFR-2 and Yes and Fyn with VEGFR-1, we observed Src and Yes kinase complex formation with VEGFR-1 upon VEGF stimulation, suggesting that at least in these tumour cells, association of SFKs with VEGFR-1 may be more promiscuous than in normal endothelial cells.

The ability of VEGF to mediate particular biologic functions appears to be cell type specific, as does expression of VEGF receptor subtypes. Many cell types exhibiting increased proliferation in response to VEGF express both VEGFR-1 and -2, or are of nonepithelial origin. The significance of aberrant expression of VEGFR-1 alone on tumours of epithelial origin remains unclear, but a recently published work found a similar lack of proliferation in VEGF-stimulated colon cancer cells expressing VEGFR-1 (Fan *et al*, 2005).

Interestingly, we observed a robust increase in cellular migration and tyrosine phosphorylation of FAK, paxillin and p130^cas^ in CRC cells with VEGF stimulation, which appears to require VEGFR-1. This is consistent with prior reports suggesting that VEGFR-1 regulates cellular migration, while VEGFR-2 mediates activation of the MAPK pathway and cellular proliferation (Barleon *et al*, 1996). Further, our finding of VEGF-induced FAK phosphorylation, resulting in its increased activation, is consistent with previous studies on VEGFR-1-overexpressing fibroblasts (Maru *et al*, 2001). This result suggests an interaction between VEGFR-1 and FAK in HT29 cells. Whether this interaction is direct or indirect remains to be determined.

**Figure 7:**
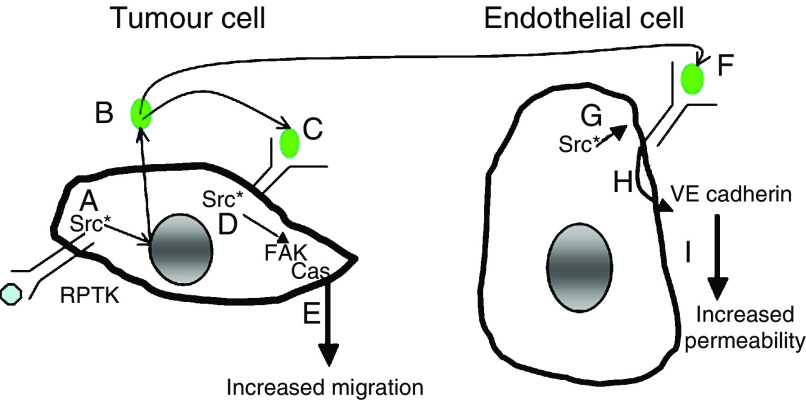
Model by which Src and VEGF contribute to tumour progression and metastasis through activities in both tumour and tumour-associated endothelial cells. In tumour cells (left) Src activation is frequent, resulting from overexpression of growth factor receptors, FAK and deregulated transcription (**A**). Src activation increases VEGF expression from tumour cells (**B**), leading to binding of VEGFR-1 (**C**), association with and further activation of Src (**D**), and the subsequent autocrine loop contributes to tumour cell migration through FAK activation (**E**). Vascular endothelial growth factor produced by tumour cells also binds VEGF receptors on endothelial cells (**F**), leading to association and activation of Src in these cells (**G**) and leading to increased permeability through VE cadherin phosphorylation affecting tumour cell extravasation (**H**), a process critical for permeability and tumour cell extravasation (**I**). Thus, inhibitors of VEGF, VEGFR-1 and Src may have therapeutic efficacy due to their effects in both normal and tumour cells.

Vascular endothelial growth factor is a pleuripotent cytokine that induces angiogenesis, proliferation, migration, differentiation and vascular permeability in endothelial cells (Leung *et al*, 1989; Millauer *et al*, 1993; Neufeld *et al*, 1999). It is secreted by most, if not all solid tumours, including those arising in colon, where it is the principal mediator of tumour angiogenesis and its expression correlates with both disease recurrence and poor prognosis (Takahashi *et al*, 1997; Werther *et al*, 2000). As anti-VEGF therapy has demonstrated efficacy in patients with advanced CRC (Hurwitz *et al*, 2004), further study of VEGF/VEGFR interactions in endothelial and, perhaps, tumour cells is warranted (Figure 7).

Here we have shown that VEGFR-1 participates in intracellular signal transduction and mediates biologic activity, specifically cellular migration, in human CRC cells. The expression of this receptor protein tyrosine kinase in tumour cells may further amplify signalling pathways already activated in colon tumour cells. Thus, VEGFR-mediated signalling offers novel targets for therapeutic interventions, either by targeting the receptor itself or important downstream mediators, such as Src. As Src activity has been shown to regulate VEGF expression, particularly under hypoxic conditions, in CRC cells (Ellis *et al*, 1998), the existence of an autocrine signalling loop capable of enhancing tumour survival and progression is intriguing. Delineating the mechanisms by which individual receptor tyrosine kinases mediate biologic activity in tumour cells is crucial for defining tumour-specific targets for optimal therapy. As recent work has demonstrated the presence of VEGFR-1 in multiple colon cancer cell lines and in both primary and metastatic tumour samples (Fan *et al*, 2005), VEGFR-1 may be a relevant clinical target, not only for its expression in endothelial cells but also for its expression/function in tumour cells.
